# Assessing Regional Cerebral Blood Flow in Depression Using 320-Slice Computed Tomography

**DOI:** 10.1371/journal.pone.0107735

**Published:** 2014-09-24

**Authors:** Yiming Wang, Hongming Zhang, Songlin Tang, Xingde Liu, Adrienne O'Neil, Alyna Turner, Fangxian Chai, Fanying Chen, Michael Berk

**Affiliations:** 1 Department of Psychiatry, Hospital Affiliated to Guiyang Medical University, Guiyang, Guizhou, China; 2 Department of Cardiology, The General Hospital of Jinan Military Region, Jinan, China; 3 Department of Neurology, First People's Hospital of Shaoyang, Shaoyang, Hunan, China; 4 Department of Cardiology, Hospital Affiliated to Guiyang Medical University, Guiyang City, Guizhou, China; 5 IMPACT Strategic Research Centre, School of Medicine, Deakin University, Geelong, Australia; 6 School of Public Health and Preventive Medicine, Monash University, Melbourne, Australia; 7 Department of Psychiatry, The University of Melbourne, Parkville, Victoria, Australia; 8 School of Medicine and Public Health, The University of Newcastle, Callaghan, New South Wales, Australia; 9 Mental Health Education And Counseling Center, Guiyang Medical University, Guiyang City, Guizhou, China; 10 Department of Psychiatry, Orygen Youth Health Research Centre, The University of Melbourne, Parkville, Victoria, Australia; 11 Florey Institute of Neuroscience and Mental Health, University of Melbourne, Parkville, Victoria, Australia; University of Electronic Science and Technology of China, China

## Abstract

While there is evidence that the development and course of major depressive disorder (MDD) symptomatology is associated with vascular disease, and that there are changes in energy utilization in the disorder, the extent to which cerebral blood flow is changed in this condition is not clear. This study utilized a novel imaging technique previously used in coronary and stroke patients, 320-slice Computed-Tomography (CT), to assess regional cerebral blood flow (rCBF) in those with MDD and examine the pattern of regional cerebral perfusion. Thirty nine participants with depressive symptoms (Hamilton Depression Rating Scale 24 (HAMD24) score >20, and Self-Rating Depression Scale (SDS) score >53) and 41 healthy volunteers were studied. For all subjects, 3 ml of venous blood was collected to assess hematological parameters. Trancranial Doppler (TCD) ultrasound was utilized to measure parameters of cerebral artery rCBFV and analyse the Pulsatility Index (PI). 16 subjects (8 =  MDD; 8 =  healthy) also had rCBF measured in different cerebral artery regions using 320-slice CT. Differences among groups were analyzed using ANOVA and Pearson's tests were employed in our statistical analyses. Compared with the control group, whole blood viscosity (including high\middle\low shear rate)and hematocrit (HCT) were significantly increased in the MDD group. PI values in different cerebral artery regions and parameters of rCBFV in the cerebral arteries were decreased in depressive participants, and there was a positive relationship between rCBFV and the corresponding vascular rCBF in both gray and white matter. rCBF of the left gray matter was lower than that of the right in MDD. Major depression is characterized by a wide range of CBF impairments and prominent changes in gray matter blood flow. 320-slice CT appears to be a valid and promising tool for measuring rCBF, and could thus be employed in psychiatric settings for biomarker and treatment response purposes.

## Introduction

In major depressive disorder (MDD), symptom development and course are influenced by brain circuits associated with vascular disease [1]. Studies conducted in depressed populations suggest that patients may exhibit impairments in regional cerebral blood flow (rCBF) and dysfunction in cortical and subcortical brain structures [2]; a close relationship has been observed between mild cognitive impairment and brain hypoperfusion [3]. rCBF is improved by treatment including cognitive-behavioral and electroconvulsive therapy (ECT) in MDD [4]–[5], suggesting a state related change. This may be linked to reduced mitochondrial energy generation and dysfunction in the disorder [6]–[7]. In parallel, there is a large literature showing alterations in brain glucose utilization in depression [8]. Therefore, monitoring rCBF in psychiatric populations and settings may be useful in the context of monitoring treatment response.

Imaging studies have previously utilized techniques such as functional magnetic resonance imaging (fMRI) and have demonstrated changes in hemodynamics of the limbic system and subcortical regions in those with depression [9]. Single-photon emission computed tomography (SPECT) has also been employed to document hypoperfusion of the frontal and prefrontal regions in late-life depressed patients [10]–[13], as well as improvements in rCBF following treatment with antidepressants [14] and ECT [15]. Similarly, MRI with arterial spin labeling has demonstrated significant CBF elevation in white matter in those for whom depression remits following antidepressant use [16].

The use of heterogeneous imaging techniques has, in some instances, yielded inconsistent results. For example, studies of depressed patients using positron emission tomography (PET) have indicated decreased glucose utilization in temporal lobe cortex [17], while others have found increased cerebral glucose metabolism in older patient populations [18]. While these studies suggest that there is a potential relationship between cognitive impairment and altered rCBF, most studies are subject to limitations including their use of semi-quantitative measurement that do not objectively reflect rCBF changes.

In contrast, the Toshiba Aquilion ONE 320 slice dynamic volume CT [19] is a refined diagnostic imaging test which utilizes dynamic volume Computed Tomography (CT) technology. It has the capacity to display soft tissue, bones and blood vessels in a singular, high resolution image and can also capture dynamic processes including blood flow and organ function, in three-dimensional real-time. This technology has advanced patient care in a range of medical settings by reducing time to diagnosis in heart disease and stroke populations. If applied in psychiatry, this technique allows for the acquisition of data pertaining to brain blood circulation and perfusion maps, including unenhanced, enhanced, delayed patterns, and assessment of arterial and venous diagrams in the same phase. It uses relatively low radiation doses and could quickly and accurately determine changes in cerebral hemodynamics as well as quantitative examination of cerebral perfusion. To date, several studies have been conducted using One 320 slice CT in coronary artery and pulmonary vascular populations [20]–[21]. While the advantages of employing whole brain CT perfusion in acute stroke, ahronic ischemia and arteriovenous malformation have been acknowledged [22], no publications investigating its utility in psychiatric populations were found at the time of writing. To our knowledge, this study will be the first to utilize 320-slice CT imaging previously used in coronary and stroke patients to assess rCBF in those with MDD and analyze the extent which it corresponds with regional cerebral blood flow velocity (rCBFV) as measured by Trancranial Doppler (TCD) ultrasound. We also aimed to compare rCBF in depressed patients to healthy control subjects to clarify regional cerebral perfusion patterns in the cerebral hemispheres, in order to explore its potential utility in psychiatric settings for possible diagnostic and treatment response purposes.

## Materials and Methods

### Subjects

The sample comprised patients (n = 39, 8 men and 31 women, aged 18–60 years, mean ages 46.74±11.42). They were hospitalized patients or outpatients with major depression seen in the Psychiatry Department of the affiliated hospital of Guiyang Medical University between April and December, 2009. Patients who met diagnostic criteria for a depressive disorder as defined by the Diagnostic and Statistical Manual of Mental Disorders-IV (DSM-IV) (diagnosed by two clinicians), had a Hamilton Depression Rating Scale 24 (HAMD24) score of >20 points, and a Self-Rating Depression Scale (SDS) score >53 were included in this study. Exclusion criteria included taking psychotropic substances or drugs influencing vessel compliance function upon enrollment (such as stimulants, hypnotics or sedatives), diseases of the nervous system (Aneurysms involving the supra-aortic vessels, chronic cerebral venous insufficiency), somatic diseases (e.g. diabetes, hypertension, coronary heart disease, atherosclerosis), or other mental disorders.

Forty one healthy volunteers, who were healthy and underwent physical examination in the physical examination center of the affiliated hospital of Guiyang Medical University between April and December 2009 were recruited concurrently as a control group. They were matched for age and gender (9 men and 32 women, aged 18–60 years, Mean age 46.22±11.68), with a HAMD24 score <20 and Hamilton Anxiety score <8, and had no history of depression, anxiety or somatic disorders.

### Ethics statement and consent

The study was approved by the Ethics of Human Investigation Committee at Guiyang Medical University (NO: 20090016) and all experiments were performed in accordance with relevant guidelines and regulations. The participants themselves or a legally authorized representative gave written informed consent to participate in the study and obtained safeguards in this study. The procedures followed were in accordance with the revised Declaration of Helsinki [23].

### Hemorheologic measures

All subjects had 3 ml of venous blood collected in the early morning (07:00–08:00) to assess blood rheology parameters (blood viscosity and hemoconcentration). Heparin was used as an anticoagulant; whole blood viscosity (including high\middle\low shear rates), hematocrit (HCT) and red blood cell sedimentation were checked [24] by an automatic blood rheometer (LBY-N6B, Beijing Precil Instrument Co. Ltd.).

### TCD screening methods

In a quiescent condition, all participants were assessed using the 2 MHz probe transcranial color-coded Doppler (TCD) sonography (Germany, DWL-X type), in accordance with Hua-Yang TCD ultrasound practices and diagnostic criteria guidelines [25]. The middle cerebral artery (MCA) and the anterior cerebral artery (ACA) were insonated through the temporal bone acoustic windows while the participant was in the conventional supine position. The vertebral artery (VA) and basilar artery (BA) were insonated through the occipital acoustic window while the participant was in the sitting position. Data were generated via the trace envelope of the measured arterial spectrum and a series of blood flow parameter values by the machine's software analyzer system. The Pulsatility Index (PI) was calculated as PI = (peak systolic velocity–end diastolic velocity)/mean blood flow velocity.

### rCBF measurement methods

Sixteen subjects (8 =  depressed, 1 men and 7 women, mean ages 43.12±12.09; 8 =  healthy, 1 men and 7 women, mean ages 44.13±10.37) were randomly selected to undergo rCBF assessment using the 320 slice CT [26] (Japan's Toshiba Aquilion ONE; non-helical scan mode, 912-channel, 16 cm coverage, lap rotation time 0.5 s, slice thickness 0.5 mm, vision 240 mm), After iodine allergy testing, participants were asked to lie in the supine position, where a 20 th catheter tube was placed in the cubital vein before the scan. A plastic tube was connected to the Empower 9900P type binocular high-pressure syringe, and participants were injected with a nonionic contrast agent (iodine Pa Alcohol Injection 37 g(I)/100 ml/bottle, Shanghai Bracco Xinyi Pharmaceutical Co. Ltd.). 50 ml and 20 ml saline was administered intravenously in the cubital fossa with an injection rate of 6 ml/s. Scan parameters were as follows: 80 kv, 100 mA. Next, brain volumes were acquired after the injection of contrast medium, scan parameters as follows: 120 kv, 200 mA, dynamic volume scanning was delayed 5 s. The time series was as follows: volume scanning was delayed 7 s, during scan across the artery was 11 s, the scan interval was 1 s, 35 s to 60 s venous and delayed scans, during every other scan interval was 5 s. A total of 19 volume data were obtained, each consisting of 320 images. Each inspection obtained a total of 6080 images (each volume data  = 0.5 min, total time  = 9.5 min). Concordant with the national standard, the total radiation dose of CT plain scan and perfusion scan was approximately 4.6 mSv.

### Perfusion image analysis methods

Nineteen volume data were imported into the perfusion fx special package for processing. The right ACA was selected as the input artery and superior sagittal sinus as an output vein. The time-density curves of the dynamic region of interest were automatically analyzed by the software. Perfusion parameters, including cerebral blood volume (CBV), CBF, mean transit time (MTT) and the time to peak (TTP), were generated using the deconvolution mathematical model. The CBV, CBF, MTT, TTP of CT perfusion images in the cross-section, sagittal and coronal plane in the whole brain were obtained by the computer pseudo-color processing. The specific hemodynamic portion of the brain in depression was obtained by drawing various regions of interests (ROIs) encompassing the regions of gray and white matter which were perfused by blood supply at every level of the ACA, MCA, and PCA, and then comparing them with the ROIs of the normal brain. The ROIs (100±5 Pix) were distributed in gray and white matter of regions of blood supply at every level of the ACA, MCA and PCA.

### Statistical analyses

Data were analyzed using SPSS Version 22.0. All measurement indicators were expressed as Means ± SEM with independent samples t-tests used to determine significant differences between the depression and control groups, where P<0.05 was considered statistically significant. Differences among groups were analyzed using one-way ANOVA. Stepwise linear regression was conducted to calculate the regression equation. A series of Pearson's correlations were undertaken to observe the strength and direction of the relationship between rCBF and rCBFV parameters. 95% confidence intervals were used in the study.

## Results

### Demographic features

No significant differences between the control and depression groups were observed for demographic variables (age, sex, blood pressure, smoking), (Table 1, 2).

**Table 1:** The comparison of demographic data between control and depression groups (Mean ± SEM).

Items	control (n = 41)	depression (n = 39)
Ages(years)	46.22±11.68	46.74±11.42
(Ranges)	18–60	18–60
Sex(M/F)(n)	9/32	8/31
Resting SBP (mm Hg)	120±25	125±27
Resting DBP (mm Hg)	80±10	76±8
Smokers/non-smokers (n)	20/21(49%/51%)	21/18 (54%/46%)

Note: no significant differences in the demographic variables (age, sex, blood pressure, smoking) between two groups, SBP: systolic blood pressure, DBP: diastolic blood pressure.

**Table 2:** The comparison of demographic data between control and depression groups using the 320 slice CT (Mean ±SEM).

Items	control (n = 8)	depression (n = 8)
Ages(years)	44.13±10.37	43.12±12.09
(Ranges)	18–60	18–60
Sex(M/F)(n)	1/8	1/8
Resting SBP (mm Hg)	118±22	120±27
Resting DBP (mm Hg)	75±10	78±8
Smokers/non-smokers (n)	1/7(13%/87%)	1/7 (13%/87%)

Note: no significant differences in the demographic variables (age, sex, blood pressure, smoking) between two groups, SBP: systolic blood pressure, DBP: diastolic blood pressure.

### Comparison of hematological parameters between the depression and control groups

Compared with the control group, whole blood viscosity (including high\middle\low shear rate) and hematocrit were significantly increased in those with depression, however no significant difference was observed between the groups for red blood cell sedimentation (Table 3).

**Table 3:** The comparison of hemorheological parameters between depression and control groups (Mean ±SEM).

Items	Control (n = 41)	Depression(n = 39)
High shear rate (mPa.s/150S^−1^)	3.92±0.31	4.63±0.39*
Middle shear rate (mPa.s/60S^−1^)	4.38±0.36	5.72±0.47**
Low shear rate (mPa.s/10S^−^)	6.43±0.70	7.75±1.10**
Hematocrit	41.32±2.91	43.34±4.68*
Red blood cell sedimentation (mm/h)	39.34±6.62	38.07±8.90

Note: Compared with control group *P<0.05, **P<0.01.

### Comparison of parameters of rCBFV between the depression and control groups

Compared with the control group, selected rCBFV parameters in the majority of cerebral arteries were decreased for those with depressive disorder (Table 4). For example, peak systolic flow velocity (Vs) and mean flow velocity (Vm) in bilateral ACA and MCA regions, and in TICA, VA and BA regions were statistically different. Further, statistically significant between group-differences showing decreased end-diastolic flow velocity (Vd) in the left ACA and the right TICA were observed. PI values were significantly lower for the depressed group in all the cerebral artery regions when compared with the control group (Table 5).

**Table 4:** The comparison of parameters of rCBFV between control and depression groups (Mean ± SEM, cm/s).

Items	Control (n = 41)	Depression(n = 39)
ACA-R-Vs	96.31±11.76	79.85±20.41**
ACA-R-Vm	63.95±8.15	54.44±14.80**
ACA-R-Vd	40.67±6.93	37.00±11.40
ACA-L-Vs	96.64±12.21	77.31±14.26**
ACA-L-Vm	64.10±7.82	52.90±11.17**
ACA-L-Vd	41.64±5.98	36.54±8.70**
TICA-R-Vs	114.03±13.44	95.87±17.40**
TICA-R-Vm	76.85±9.69	69.11±21.98*
TICA-R-Vd	51.28±7.18	46.46±10.30*
TICA-L-Vs	112.54±12.32	97.69±20.41**
TICA-L-Vm	75.28±9.16	67.90±15.47*
TICA-L-Vd	50.46±7.22	47.85±12.17
MCA-R-Vs	117.05±13.87	98.15±17.60**
MCA-R-Vm	78.97±10.24	68.62±12.39**
MCA-R-Vd	52.95±7.46	47.87±10.15*
MCA-L-Vs	114.59±12.94	98.10±20.17**
MCA-L-Vm	77.31±9.51	68.72±14.69**
MCA-L-Vd	51.72±7.70	48.46±11.41
VA-R-Vs	71.85±10.06	61.87±11.68**
VA-R-Vm	48.49±6.55	43.87±8.42**
VA-R-Vd	32.62±4.42	30.64±6.82
VA-L-Vs	72.59±8.93	62.79±10.77**
VA-L-Vm	49.67±6.04	45.08±7.56**
VA-L-Vd	33.49±4.18	32.08±5.56
BA-Vs	71.85±10.06	67.82±11.68**
BA-Vm	52.59±6.57	48.21±9.06*
BA-Vd	35.38±4.97	34.12±7.23

Note: compared to the control group, *P<0.05, **P<0.01.

rCBFV: regional cerebral blood flow velocity.

MCA: middle cerebral artery, ACA: anterior cerebral artery.

PCA: posterior cerebral artery, TICA: the tip of internal carotid artery.

VA: vertebral artery, BA: basilar artery, L: Left, R: Right,

Vs: systolic peak velocity, Vm: mean flow velocity, Vd: diastolic velocity.

**Table 5:** The comparison of the values of PI between control and depression groups (Mean±SEM).

Items	Control (n = 41)	Depression(n = 39)
ACA-R	0.89±0.10	0.80±0.18*
ACA-L	0.86±0.79	0.78±0.15*
TICA-R	0.88±0.08	0.76±0.12**
TICA-L	0.87±0.09	0.75±0.12**
MCA-R	0.82±0.08	0.74±0.11*
MCA-L	0.82±0.09	0.73±0.11*
VA-R	0.83±0.11	0.72±0.10**
VA-L	0.79±0.09	0.68±0.09**
BA	0.77±0.10	0.70±0.11*

Note: Compared with control group *P<0.05, ** P<0.01.

PI: Pulsatility index, ACA: anterior cerebral artery,

TICA: the tip of internal carotid artery, MCA: middle cerebral artery,

VA: vertebral artery, BA: basilar artery, L: Left, R: Right.

### Comparison of rCBF between the depression and control group

Compared with the control group, the values of rCBF in bilateral gray and white matter of the cerebral hemispheres were lower for those with a depressive disorder; with the exception of white matter in the left and right PCA region and white matter of the right MCA region (Table 6).

**Table 6:** The comparison of rCBF between control and depression groups (Mean ±SEM, ml·min-1·100 g-1, n = 8).

Items	Control	Depression
ACA-R-GM	46.11±12.41	36.96±10.20*
ACA-L-GM	44.72±10.95	36.45±11.67*
ACA-R-WM	23.74±6.74	20.20±5.71*
ACA-L-WM	25.21±6.63	21.67±6.68*
MCA-R-GM	49.63±10.78	44.09±12.76*
MCA-L-GM	49.71±15.61	41.19±13.77**
MCA-R-WM	25.05±6.94	21.97±5.71
MCA-L-WM	26.17±6.91	22.52±5.75*
PCA-R-GM	46.63±10.06	39.02±10.92*
PCA-L-GM	45.76±11.91	34.32±12.21**
PCA-R-WM	27.73±11.44	24.73±8.61
PCA-L-WM	28.33±10.85	25.65±8.45

Note: Compared with control group * P<0.05, ** P<0.01.

rCBF: regional cerebral blood flow, ACA: anterior.

cerebral artery, MCA: middle cerebral artery.

PCA: posterior cerebral artery, L: Left, R: Right.

GM: gray matter, WM: white matter.

### Comparison of rCBF between left and right cerebral hemisphere in depression

**Figure 1:**
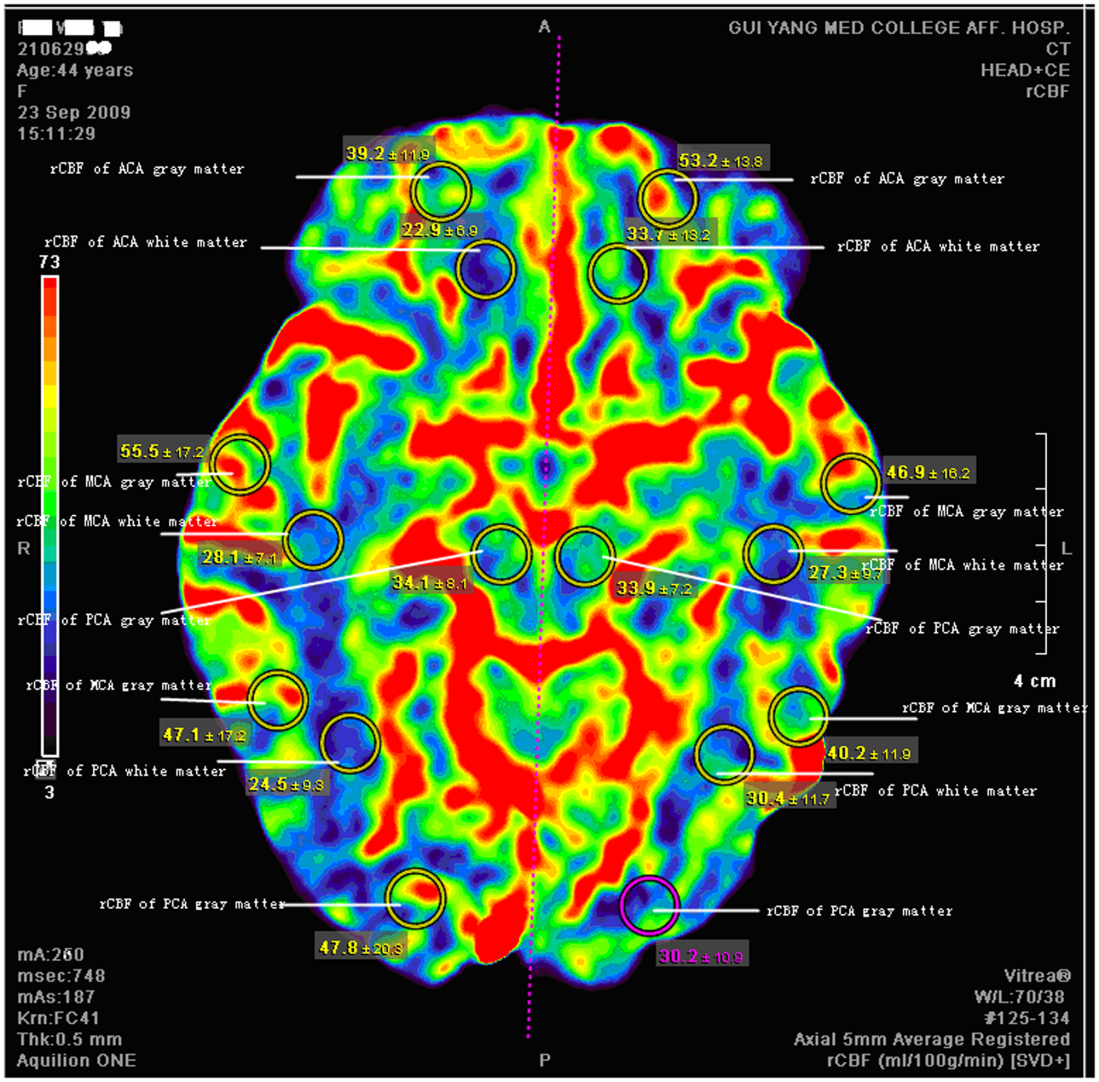
Case example: Coronal slices from dynamic CT angiography taken from a 44-year-old woman with depression. Circles represent regions of interest, with the mean ± SEM from 19 volume data presented. In MCA (middle cerebral artery) regions, the values of rCBF (regional cerebral blood flow) in left gray matter were lower than in the right [Left: 46.9 and 40.2 ml/(min·100 g),Right: 55.5 and 47.1 ml/(min·100 g)]. In PCA (posterior cerebral artery) regions, the values of rCBF in left gray matter were lower than in the right [Left: 30.2 ml/(min·100 g), Right: 47.8 ml/(min·100 g)].

Compared with the right cerebral hemisphere, rCBF values in left gray matter in MCA and PCA regions were significantly lower in those with depressive disorder (Table 7, Figure 1).

**Table 7:** The comparison of rCBF between the left and right cerebral hemispheres in depression group (Mean ±SEM, ml·min-1·100 g-1, n = 8).

Items	Left	Right
ACA -GM	36.45±11.67	36.96±10.20
ACA -WM	21.67±6.67	20.21±5.71
MCA-GM	41.49±13.77*	44.09±12.77
MCA-WM	22.52±5.75	21.97±5.71
PCA-GM	34.32±12.21*	39.02±10.92
PCA-WM	25.64±8.45	24.73±8.61

Note: Compared with Right * P<0.05.

rCBF: region cerebral blood flow, ACA: anterior cerebral artery.

MCA: middle cerebral artery, PCA: posterior cerebral artery.

L: Left, R: Right, GM: gray matter, WM: white matter.

### The relationship between rCBF and rCBFV in MDD group

**Figure 2:**
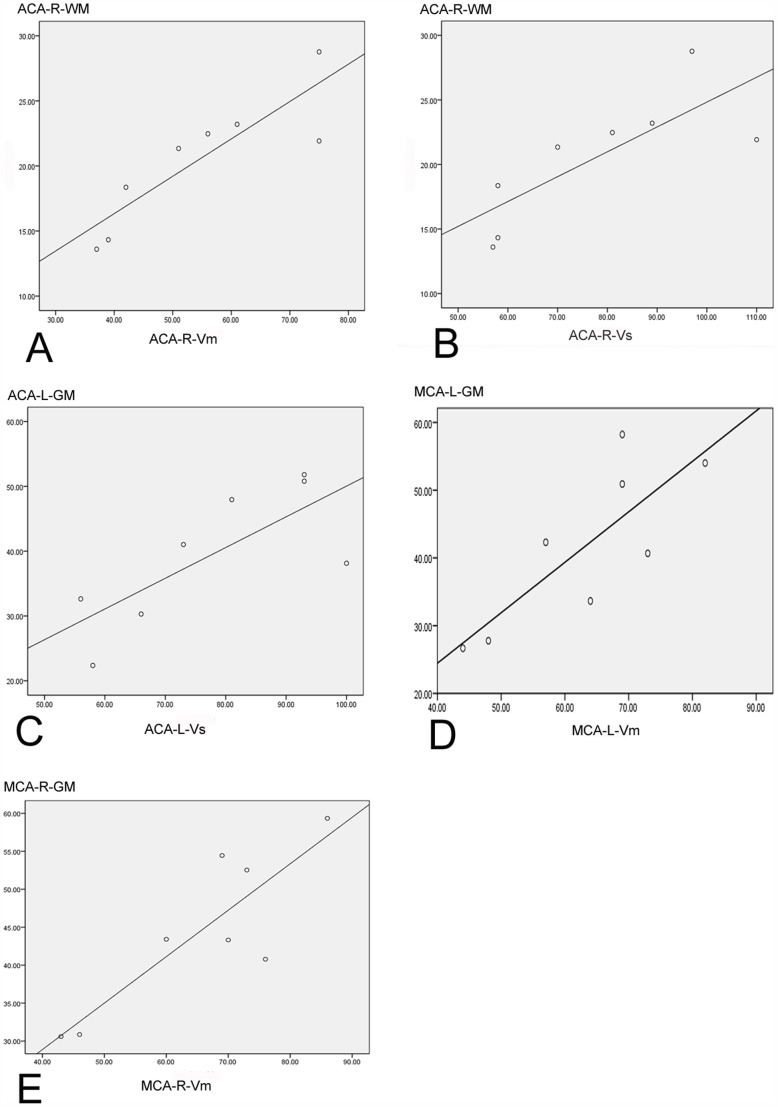
The correlation between rCBFV and rCBF in cerebral artery regions. A: A positive correlation between mean flow velocity (Vm) and rCBF in white matter (WM) of right ACA (anterior cerebral artery) (P<0.01; r = 0.874). B: A positive correlation between systolic peak velocity (Vs) and rCBF in WM of right ACA (P<0.01; r = 0.778). C: A positive correlation between Vs and rCBF in gray matter (GM) of left ACA (P<0.05; r = 0.758). D: A positive correlation between Vm and rCBF in GM of left MCA (middle cerebral artery) (P<0.05; r = 0.802). E: A positive correlation between Vm and rCBF in GM of right MCA (P<0.01; r = 0.859). rCBFV: regional cerebral blood flow velocity, rCBF: region cerebral blood flow.

When exploring the strength and direction of the association between rCBF and rCBFV in the MDD group, we found a positive correlation for gray matter of the bilateral ACA and MCA regions, except for the right ACA (Vs, Vm, Vd) and left ACA (Vd); and in white matter of the right ACA (Vs, Vm, Vd) and right MCA (Vm). The strongest correlation coefficients were produced for right ACA (Vm r = 0.874, Vd r = 0.839 for white matter) and right MCA Vm (r = 0.859 for gray matter), left MCA (Vs r = 0.760, Vm r = 0.802, Vd r = 0.748 for gray matter). Overall, the strength of the relationships were most consistent for gray matter. The correlation coefficients are displayed in Table 8, the regression equation between rCBFV and rCBF in Table 9 and illustrated in Figures 2.

**Table 8:** The correlation coefficients between rCBF and rCBFV in depression group (n = 8).

rCBFV items	rCBF of Gray matter	rCBF of White matter
ACA-R-Vs	0.599	0.778*
ACA-R-Vm	0.674	0.874**
ACA-R-Vd	0.624	0.839**
ACA-L-Vs	0.758*	0.607
ACA-L-Vm	0.720*	0.568
ACA-L-Vd	0.575	0.569
MCA-R-Vs	0.706*	0.520
MCA-R-Vm	0.859**	0.718*
MCA-R-Vd	0.748*	0.683
MCA-L-Vs	0.760**	0.426
MCA-L-Vm	0.802**	0.535
MCA-L-Vd	0.748**	0.538

Note: The correlation coefficient t test * P<0.05. ** P<0.01.

rCBFV: regional cerebral blood flow velocity, rCBF: region cerebral blood flow.

ACA: anterior cerebral artery, MCA: middle cerebral artery, L: Left, R: Right.

Vs: systolic peak velocity, Vm: mean flow velocity, Vd: diastolic velocity.

**Table 9:** Regression equation between rCBFV and rCBF in depression group (n = 8).

Regression equation	*P* value
ACA-L-GM-rCBF = 2.636+0.474(ACA-L-Vs)	0.029
ACA-R-WM-rCBF = 4.852+0.287(ACA-R-Vm)	0.005
ACA-R-WM-rCBF = 7.831+0.86(ACA-R-Vm)-0.441(ACA-R-Vs)	0.003
MCA-L-GM-rCBF = 0.745(MCA-L-Vm)-5.333	0.017
MCA-R-GM-rCBF = 4.420+0.612(MCA-R-Vm)	0.006

rCBFV: regional cerebral blood flow velocity, rCBF: region cerebral blood flow.

ACA: anterior cerebral artery, MCA: middle cerebral artery, L: Left, R: Right.

GM: gray matter, WM: white matter, Vs: systolic peak velocity, Vm: mean flow velocity.

## Discussion

For the first time to our knowledge, we have provided data on the potential utility and novel application of the 320 slice Dynamic volumes CT in a clinically depressed population, a tool validated in coronary and stroke populations. These data demonstrate that patients with depression are characterized by a wide range of CBF impairments, in particular prominent changes in gray matter, when compared with healthy controls. rCBF data generated using this technology in a sub-sample of MDD patients was shown to correlate well with those generated by other measures (TCD ultrasound), suggesting that it could be employed in psychiatric settings for potential diagnostic and treatment response purposes.

Previous studies have suggested reduced rCBF in MDD, illustrated by hypoperfusion in the frontal lobe, temporal lobe, and in the limbic system [27]–[28]. Indeed, our results are consistent with other functional imaging studies conducted in this area using SPECT imaging [29], [12] and a range of other techniques. As these techniques are often hampered by limitations including accessibility, poor resolution and subjectivity, this study provides some support for the use of CT perfusion imaging for rapid imaging and assessment to determine the CBF dynamics quickly, accurately and in 3 dimensional high resolution in a psychiatric setting [30]–[31]. The resulting image aligns with real data [32]. Therefore, the use of 320-slice CT for the objective study of cerebral perfusion appears to have clinical value potentially supplanting older techniques.

We found that there was decreased rCBFV in the majority of cerebral arteries in depressive patients, for example, Vs and Vm differed in the bilateral ACA, CA, TICA, VA and BA regions, while Vd differed in the left ACA and right TICA regions. Furthermore, in patients with depression, there was a positive relationship between the rCBFV and the corresponding vascular rCBF in the gray matter of bilateral ACA and MCA regions (with the exception of right ACA (Vs, Vm, Vd) and left ACA (Vd)), and the white matter of the right ACA (Vs, Vm, Vd) and MCA (Vm) regions. We also demonstrated that the rCBF values of left gray matter in MCA and PCA regions were lower than that of right in those with depressive disorder. Hardoy's results [33], in MDD showing hypoperfusion in the left frontal and temporal areas and perfusion asymmetry, was consistent with the findings in our cohort.

A reduction in blood flow could be due to either functional or structural deficiencies. TCD PI reflects cerebrovascular resistance by the flow velocity waveform [34]. Kwater et al. [35] reported a strong association between higher PI and systemic arterial stiffness in patients with atherosclerosis, however atherosclerosis reduces cerebrovascular reactivity, leading to higher PI values. In our study, however, PI was decreased in the depressive group compared with controls, suggesting that the reason for the reduced blood flow in the depressive group may be functional rather than structural.

We also observed that the whole blood viscosity and hematocrit were significantly increased in the depressed patients. Blood viscosity is a measure of the thickness and stickiness of blood, and increased levels of blood viscosity has been associated with arterial disease including myocardial infarction and stroke [36]–[37]. It has been suggested that stress is associated with hemoconcentration of the cerebral hemispheres in patients with MDD [38]. Lechin [39] argued that blood viscosity is positively involved with the levels of neural sympathetic and cholinergic activity in MDD, while Wong et at. found that decreased blood viscosity is correlated with the improvement of depressive symptoms after antidepressant treatment in MDD.

There is evidence of significant hypoperfusion in the gray matter of the cerebral hemispheres in people with depression [40]. The reason for this may be concordant with the proximal relationship between the dominant hemisphere cortex and human cognition, emotion and related functions, while its relationship to deep white matter appears more peripheral. Botteron [41] reported that elderly depressed people with memory impairment showed changes in the medial temporal lobe and its adjacent structures.

Su liang et al [42] proposed that elderly depressive patients with cognitive impairment exhibited decreased local glucose metabolism in the caudate nucleus bilaterally, the inferior frontal gyrus, left cingulate and anterior central gyrus regions, and the decline in both executive function and memory function was related to low local glucose metabolism in the caudate nucleus bilaterally, the frontal lobe, temporal lobe, the left central gyrus and limbic brain regions of deep white matter. Indeed, our results were concordant with this study.

### Limitations of both the technique and of the study

The specific hemodynamic regions of the brain in depression was obtained by drawing various regions of interests encompassing the regions of gray and white matter which were perfused by ACA, MCA, and PCA blood supply, and then comparing them with the ROIs of the normal brain. This captured representative regional cerebral blood flow, but not the actual value of the lobes of the brain. Secondly, because relatively few subjects had rCBF assessment, this could result in possible bias. While changes in rCBF were seen, potential clinical applications require further study. Lastly a multivariate ANOVA analysis testing multiple correlations was not performed, and differences between groups were analyzed using one-way ANOVA.

### Conclusions

These data suggest that people with depression are characterized by a wide range of cerebral blood flow impairments and there appear to be more prominent changes in left gray matter. The 320 slice Dynamic volumes CT appears to be a potentially useful tool for measuring rCBF, with advantages over existing instruments. This technique could be employed in psychiatric settings for biomarker, diagnostic and treatment response purposes. Future studies should replicate this study in a larger sample, acquiring additional data to determine the factors influencing blood supply to the region of the brain of patients affected in those with depression. The relationship to treatment response in particular needs to be explored.
